# Protective effects of resveratrol on the expression of *catalase, glutathione peroxidase*, and *superoxide dismutase* genes in the ovary and their activity in the serum of rats exposed to lead acetate: An experimental study

**DOI:** 10.18502/ijrm.v22i11.17821

**Published:** 2025-01-10

**Authors:** Mohammad Karimian, Mozhdeh Ghadiri, Seyedeh Mahsa Poormoosavi, Hossein Najafzadehvarzi

**Affiliations:** ^1^Department of Molecular and Cell Biology, Faculty of Basic Sciences, University of Mazandaran, Babolsar, Iran.; ^2^Faculty of Pharmacy, Ayatollah Amoli Branch, Islamic Azad University, Amol, Iran.; ^3^Department of Histology, School of Medicine, Dezful University of Medical Sciences, Dezful, Iran.; ^4^Cellular and Molecular Biology Research Center, Health Research Institute, Babol University of Medical Sciences, Babol, Iran.

**Keywords:** Resveratrol, Lead acetate, Oxidative stress, Ovary, In silico, Rat.

## Abstract

**Background:**

Lead (Pb) could be toxic to the female reproductive system, and resveratrol (Res) may overcome this toxicity.

**Objective:**

To investigate the Res impact on the catalase (*Cat*), glutathione peroxidase (*Gpx*), and superoxide dismutase (*Sod*) gene expression in the ovary and on the Cat and Gpx enzyme activity in the serum of rats exposed to lead acetate.

**Materials and Methods:**

In this experimental study, 33 female Wistar rats (8–10 wk, 180–200 gr) were divided into 6 groups: a control group (normal saline), a Res group (40 mg/kg), and a Pb group (lead acetate 30 mg/kg). 3 additional groups received lead acetate (30 mg/kg) with Res at 20, 40, and 80 mg/kg for 21 days. Gene expression of *Cat*, *Gpx*, and *Sod* was measured via qPCR, and serum Cat and Gpx activity was assessed using standard methods. Bioinformatics tools were used to evaluate Res effects on gene and protein function.

**Results:**

Lead acetate significantly downregulates *Cat*, *Gpx*, and *Sod* gene expression, but Res significantly upregulates gene expression, especially at doses of 40 mg/kg for *Cat*, 20 mg/kg and 40 mg/kg for *Gpx*, and 80 mg/kg for *Sod*. Cat and Gpx enzyme activity increased and decreased in the lead acetate group, respectively. However, Res in all doses decreased only the Cat enzyme activity. Bioinformatics analysis indicates that Res can interact with the promoter regions and cavities of all 3 enzymes.

**Conclusion:**

Pb can dysregulate the expression and activity of the studied enzymes. However, the impact of Res is influenced by the dose, with 40 mg/kg frequently being the most effective.

## 1. Introduction 

The lead (Pb) could target major tissues including the kidneys, liver, nervous system, etc. Also, this heavy metal may have detrimental effects on the reproductive system. Pb results in impaired male fertility in humans and rodents (1). Moreover, it may cause female fertility problems such as ovarian disorders, premature delivery, miscarriage, and infant mortality (2). Animal studies show that exposure to Pb disrupts steroidogenesis, alters the estrous cycle, and reduces the number of corpus luteum (3).

Although the molecular pathogenesis mechanism of Pb has not been completely elucidated, the oxidative stress pathway has recently been considered. Oxidative stress occurs when there is an imbalance between antioxidants and pro-oxidants. This imbalance may be caused by the production of reactive oxygen species (ROS) and nitrogen species, possibly by inhibiting the δ -aminolevulinic acid dehydratase, an enzyme involved in heme synthesis and is very sensitive to Pb (4). It can also be caused by decreased antioxidant defenses such as glutathione peroxidase (*Gpx*), superoxide dismutase (*Sod*), catalase (*Cat*), and so forth. ROS could affect several physiologic functions of the ovary, such as oocyte maturation, ovarian steroid genesis, ovulation, implantation, formation of blastocysts, luteolysis, and luteal preservation in pregnancy (5).

Nowadays, herbal medicine has been explored to overcome the detrimental effects of oxidative stress. Resveratrol (Res), a plant-derived polyphenol found abundantly in grape seeds and skins, exhibits outstanding antioxidant properties (6). Some previous studies have been conducted on the antioxidant effects of Res. For example, one study showed that Res prevents ethanol-induced oxidative stress in the hippocampus by decreasing cellular lipid peroxidation but does not inhibit the Cat or Sod activation. However, it enables glutathione to remain active and maintain sufficient concentrations in its reduced form (7). It was also reported that Res may be known as an effective protective agent for neural stem cells because it reduces stress by enhancing the activity of antioxidant enzymes, reducing nitric oxide production, inhibiting nitric oxide synthase, and reducing damage to nuclear and mitochondrial DNA (4). In addition, it was found that Res can reduce intestinal damage by improving oxidative status (8). A recent study also investigated the simultaneous effects of lead and Res on *Drosophila melanogaster* and found that Res effectively protected *Drosophila melanogaster* from Pb toxicity (9).

As it is known, the antioxidant effects of Res against lead toxicity are limited, and according to our knowledge, such a study has not been done in ovarian tissue at all. Therefore, this study aimed to investigate the effects of Res on the expression of *Cat, Gpx,* and *Sod* genes in the ovary and on the activity of Cat and Gpx enzymes in the serum of rats exposed to lead acetate.

## 2. Materials and Methods

### Chemicals

The materials used in this research include lead acetate (Sigma-Aldrich, St. Louis, MO, USA), Resveratrol (Sigma-Aldrich, St. Louis, MO, USA), ketamine (Sigma-Aldrich, St. Louis, MO, USA), xylazine (Sigma-Aldrich, St. Louis, MO, USA), Gpx measurement kit (Nagpix^TM^ Kit, Navand Salamat Co., Urmia, Iran), RNA extraction kit (Pars-Tous, Mashhad, Iran), cDNA synthesis kit (Pars-Tous, Mashhad, Iran), real-time polymerase chain reaction (PCR) related items (Pishgam, Tehran, Iran), SYBR Green 2X Mix (SinaClon, Tehran, Iran), and primers for PCR (Pishgam, Tehran, Iran).

### Animals

33 female Wistar rats (8–10 wk, 180–200 gr) were enrolled in this experimental study. The rats were obtained from the Animals Breeding and Experimental Research Center of Babol University of Medical Sciences, Babol, Iran. Rats were exposed to enough food and water under controlled conditions including humidity 55–60%, temperature 23 ± 2 C, and 12 hr light/dark cycle.

### Experimental design

The rats were randomly divided into 6 groups. 1) control group treated with a daily intraperitoneal (I.P.) injection of normal saline (C, n = 6), 2) Pb group treated with 30 mg/kg Pb acetate (Pb, n = 6), 3) Res group treated with 40 mg/kg Res (R, n = 6), 4) Pb + R (L) group simultaneously treated with Pb acetate + 20 mg/kg Res (n = 5), 5) Pb + R (M) group simultaneously treated with Pb acetate + 40 mg/kg Res (n = 5), 6) Pb + R (H) group simultaneously treated with Pb acetate + 80 mg/kg Res (n = 5).

The acute I.P. LD50 for Pb acetate in rodents is reported to be 100–200 mg/kg (10). In addition, the doses of Pb acetate and Res were selected according to the previous literature (11).

**Figure 1:**
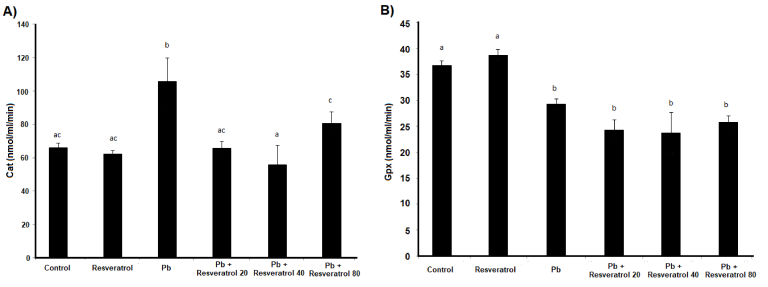
Flowchart of study. The stages of the experiment from grouping to treatment and related experiments are summarized in the figure. Ctr: Control, Res: Resveratrol, Pb: Lead acetate, I.P.: Intraperitoneal injection, *Cat*: Catalase, *Gpx*: Glutathione peroxidase, *Sod*: Superoxide dismutase.

Treatments were performed for 21 days. Pb acetate was injected I.P., and Res was administered daily by gavage. Different groups were considered to investigate the effects of lead and Res as well as their interaction compared to the control group without intervention. The rats were anesthetized with 100 mg/kg and 10 mg/kg doses of ketamine and xylazine, respectively. The right and left ovaries were resected and rapidly transferred to -80 C and formalin for further molecular and histopathological tests, respectively. The selection process of rats is presented in figure 1.

### Measuring the activity of antioxidant enzymes

After blood collection and centrifugation of samples at 3000 RPM for 10 min, blood serum was separated. Cat activity in the serum was measured using Goth's method (1991). Briefly, 0.1 mL of serum was incubated with 1 mL of a reaction mixture containing 50 mM potassium phosphate buffer (pH 7.0) and 10.6 mM hydrogen peroxide (H_2_O_2_) at 37 C for 60 sec. The reaction was stopped by adding 0.5 mL of 32.4 mM ammonium molybdate solution, forming a yellow complex with H_2_O_2_. The absorbance of the yellow color was measured at 405 nm using a spectrophotometer (Unico 2100), with a blank containing distilled water instead of serum. One unit of Cat activity was defined as the amount of enzyme that decomposes 1 mol of H_2_O_2_ per minute (12).

The activity of serum Gpx was measured using the Nagpix^TM^ kit (Navand Salamat Co., Urmia, Iran). Gpx are enzymes that protect organisms from oxidative damage. They reduce lipid hydroperoxides to alcohols or hydrogen peroxide to water, converting reduced glutathione (GSH) into oxidized glutathione (GSSG). Gpx reduces cumene hydroperoxide by oxidizing GSH to GSSG, and then GSSG is regenerated to GSH by glutathione reductase, using NADPH. The NADPH consumed in this process serves as a marker to assess Gpx levels, with Gpx activity measured at 340 nm.

### Real-time quantitative PCR analysis

The real-time PCR method was used to evaluate the expression changes of genes involved in oxidative stress. Total RNA from the ovary of all rats was isolated using a commercial kit according to the manufacturer's instructions. The quantity and quality of extracted RNA were assessed using a NanoDrop spectrophotometer (Thermo Scientific NanoDrop 2000/2000c, USA) and 1% agarose gel electrophoresis, respectively. The cDNA synthesis was performed using a kit from the same company. Real-time quantitative PCR was carried out in a 20 μ l final volume containing 10 μ l SYBR Green 2X Mix, 0.5 μ l of each forward and reverse primers (stock: 10 pmol/ μ l), and 1 μ l of diluted cDNA (based on concentration). The *Gapdh* gene was used as an internal control in real-time procedures. All real-time PCR and agarose gel reagents and chemicals were purchased from SinaClon Co. The real-time PCR was performed using an ABI StepOnePlus^TM^ thermal cycler (Applied Biosystems, USA). The sequence of designed specific primers by Oligo7 software and annealing temperatures are detailed in table I. The data were normalized based on Gapdh expression levels, and the relative changes in expression were determined using the 2 ${\;}^{-\Delta \Delta \text{Ct}}$ calculation method.

### Bioinformatics analysis 

2 approaches were used to evaluate the effect of Res on Cat, Gpx, and Sod enzymes. The first approach examined the effect of Res on the promoters of these genes, while the second assessed Res's direct interaction with the enzyme structures. To evaluate the interaction of Res with promoter regions, at first, the sequence of promoters for 3 *Cat*, *Gpx*, and *Sod* genes was retrieved from the Eukaryotic Promoter Database server. 3 potential Res DNA binding sequences, CCAATTGG, AATT, and TTAA, were considered upstream of these genes (13).

For assessment of the interaction of Res with the Cat, Gpx, and Sod enzymes, at first, the protein sequences of these proteins were obtained from Expasy databases. Then the 3D structure of proteins was predicted by Phyre2 webserver. In addition, the structure of Res was deduced from PubChem (CID: 445154). Then, the 3D structure of proteins in combination with Res was analyzed using PyRx software. The interaction of each abovementioned enzyme with Res was evaluated using Molegro Virtual Docker after the required treatments and corrections.

**Table 1:** Primer sequences, PCR product size, and annealing temperatures in real-time PCR

**Gene**	**Accession No.**	**Primer sequence (5 '→ 3 ' )**	**AT ºC**	**Size (bp)**
*Cat*	NM_012520	F: TCCATCCTTTATCCATAGCC R: TTAACCAGCTTGAAGGTGTG	58	189
*Gpx*	NM_030826	F: GCGTCCCTCTGAGGCACCAC R: AAGTTGGGCTCGAACCCACC	59	191
*Sod*	NM_017050	F: TGTGATCTCACTCTCAGGAG R: CTCAGACCACATAGGGAATG	58	178
*Gapdh*	NM_017008	F: GGCAAGTTCAACGGCACAG R: CGCCAGTAGACTCCACGAC	57	142
PCR: Polymerase chain reaction,* Cat*: Catalase, *Gpx*: Glutathione peroxidase, *Sod*: Superoxide dismutase, *Gapdh*: Glyceraldehyde-3-phosphate dehydrogenase, AT: Annealing temperature, bp: Base pair				

### Ethical Considerations

In this study, all ethical guidelines related to working with laboratory animals were followed. All interventions in rats were conducted according to the protocol provided by the Ethics Committee of the Babol University of Medical Sciences, Babol, Iran (Code: IR.IAU.AMOL.REC.1400.058).

### Statistical Analysis 

All data for enzyme activity and gene expression were expressed as mean ± standard error and mean ± standard deviation, respectively. Biochemical and gene expression comparisons between groups were evaluated by using one-way analysis of variance (ANOVA) followed by Tukey's post hoc test. SPSS version 20 (SPSS Inc., IBM Corp, Armonk, NY, USA) was employed for statistical analysis of the data. A p-value < 0.05 was considered statistically significant.

## 3. Results

### Biochemical assay 

**Figure 2:**
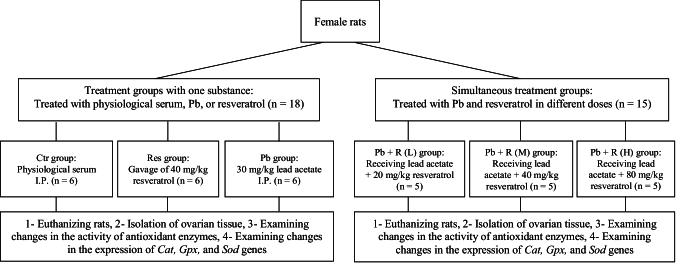
Biochemical results. A) Mean ± SEM of Cat activity in different groups. B) Mean ± SEM of Gpx activity in different groups. Dissimilar letters indicate significant differences between these groups (p < 0.05). Cat: Catalase, Gpx: Glutathione peroxidase, Pb: Lead.

The examination of Cat activity showed that lead acetate increased the activity of this enzyme, with this parameter being 65.79 nmol/min/ml in the control group and 105.77 nmol/min/ml in the lead acetate group. This difference was statistically significant (p = 0.005). The use of Res at doses of 20 mg and 40 mg significantly reduced Cat activity (Figure 2A). For Gpx enzyme activity, it was shown that with Pb administration, the activity of this enzyme significantly decreased compared to the control group, with the enzyme activity level in the control group at 36.77 nmol/min/ml and in the lead acetate group reduced to 29.25 nmol/min/ml. Treatment with Res at doses of 20 mg, 40 mg, and 80 mg did not Pb to significant changes in Gpx activity compared to the lead acetate group (Figure 2B).

### Influence of Pb and Res on *Cat*, *Gpx*, and *Sod* gene expression 

**Figure 3:**
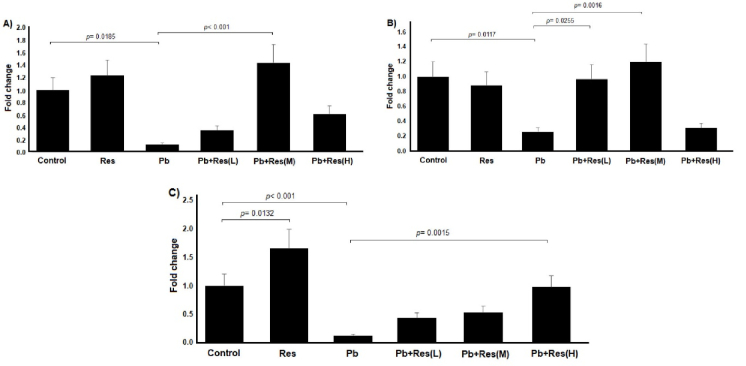
Relative expression of *Cat*, *Gpx*, and *Sod *genes in different groups. Changes in the expression of A) *Cat*, B) *Gpx*, and C) *Sod* genes due to the administration of Pb and different doses of Res. Pb + R (L): Pb + Res (20 mg/kg), Pb + R (M): Pb + Res (40 mg/kg), Pb + R (H): Pb + Res (80 mg/kg). Res: Resveratrol, Pb: Lead, L: Low dose, M: Medium dose, H: High dose.

A significant downregulation of *Cat* was observed in the ovary of Pb-treated rats compared to the control group (p = 0.0185). Co-treatment with Pb and Res (40 mg/kg) resulted in the upregulation of the *Cat* compared to the Pb group (p < 0.001). The results of gene expression are demonstrated in figure 3A. As shown in figure 3B, the expression of *Gpx* was significantly decreased in the Pb-treated group (p = 0.0117). Administration of Res (20 and 40 mg/kg) significantly upregulated *Gpx* gene expression in rat ovaries (Figure 3B). Similar to the other 2 genes, *Sod* expression decreased in the rat ovaries due to Pb induction. The expression of *Sod* was increased after the co-administration of Res (80 mg/kg) and Pb (p = 0.002). Also, the expression of *Sod* in the Res group was surprisingly increased compared to the control group (p = 0.0131) (Figure 3C).

### In silico analysis

In this study, we first evaluated the effects of Res on the promoter region of *Cat*, *Sod*, and *Gpx*. We found that there were no “CCAATTGG” DNA sequences for up to 1000 nucleotides upstream of the start codon for any of the 3 genes. However, analysis of the upstream regions for AATT and TTAA sequences revealed that there are 10 binding sites for Res upstream of *Cat*, 4 binding sites for *Gpx*, and 10 binding sites for *Sod*.

**Figure 4:**
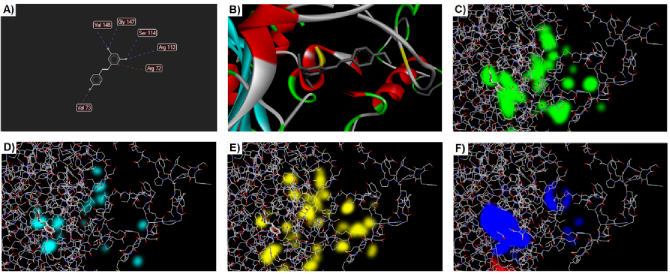
Docking results for Cat enzyme. The ligand map shows the interaction of 6 residues A) The proximity of the studied pose to the amino acid cysteine is shown in yellow. B) Steric, C) Hydrogen acceptor, D) Hydrogen donor, E) Electrostatic, and F) Regions are demonstrated by different colors.

After docking analysis, we found 5 poses for the interaction of Cat and Res. For the first pose, there is a total MolDock score equal to -75.189. Based on the data deduced from the docking software, most of these energies were related to hydrogen bonds and some were related to steric interactions. This pose formed 3 hydrogen bonds with the glycine 147, valine 146, serine 114, arginine 112, and valine 73 of the enzyme Cat. Also, arginine 72 established one steric bound with Res (Figure 4).

**Figure 5:**
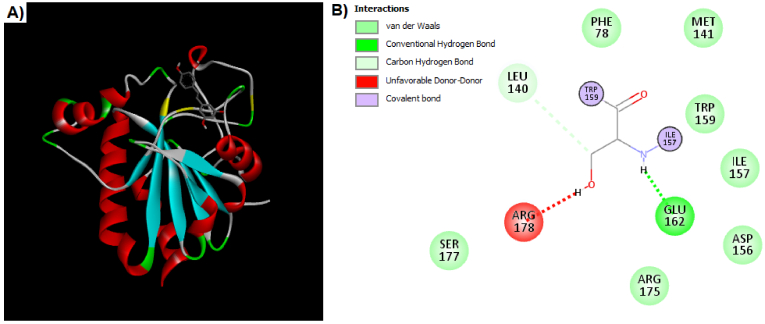
A) Docking results for interaction of Res with Gpx enzyme. This interaction could happen near the cysteine residues that are shown in yellow. B) The Res has some interactions such as van der Waals, hydrogen bonds, and covalent bonds with Gpx enzyme.

For analysis of interaction between Res and Gpx, enzymes were analyzed by PyRx software and the search was done in a grid box with center-x: -3.4727, center-y: 12.7757, and center-z: 34.1178 with dimensions size-x: 11.8893, size-y: 16.3107, and size-z: 23.5655. The value of exhaustiveness was considered as 8. The best-scoring pose was analyzed using the Discovery Studio Visualizer. The binding affinity for the best pose was calculated at -4.7 kcal/mol. As depicted in figure 5, the Res is positioned near cysteine residues. In addition, the Res could interact with Gpx by van der Waals, hydrogen bonds, and covalent bonds.

**Figure 6:**
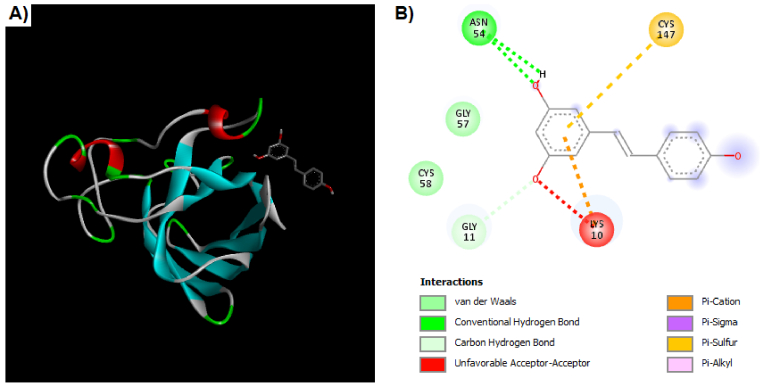
A) Docking results for interaction of Res with Sod enzyme. This position of Res is in a surface cavity of the molecule. B) The Res has some interactions such as van der Waals, hydrogen bonds, Pi-Cation, Pi-Sigma, Pi-Sulfur, and Pi-Alkyl with Sod enzyme.

The interaction of the Res and Sod enzymes were evaluated similarly to the Gpx enzyme. The grid box coordinates were center-x: 16.1511, center-y: -6.1511, and center-z: 15.3738 with dimensions size-x: 15.1553, size-y: 15.2744, and size-z: 15.5647. After the docking process, 9 poses were determined by PyRx that the first pose had a binding affinity of -4.1 kcal/mol. The theoretical binding mode of the Res in the binding site of the Sod enzyme was shown in figure 6. The Res was surrounded by the residues Cys 147, Cys 58, Gly 57, Asn 54, Gly 11, and Lys 10. As depicted in figure 6, the Res could interact with Sod by van der Waals, hydrogen bonds, sulfur bonds, and others.

## 4. Discussion

In this study, we evaluated the effects of Res on the expression of *Cat*, *Gpx*, and *Sod* genes and *Cat*, *Gpx* activity in the ovaries of rats treated with Pb acetate. We found that lead acetate reduces the expression of *Cat*, *Gpx*, and *Sod* genes, while Res increases their expression, especially at 40 mg/kg for *Cat*, 20 mg/kg and 40 mg/kg for *Gpx*, and 80 mg/kg for *Sod*. Cat activity increased, and Gpx activity decreased in the lead acetate group, but Res only reduced Cat activity.

The Sod, Gpx, and Cat enzymes protect cells from oxidative damage by reducing the effect of oxidant molecules in tissues. Due to their high ability to neutralize free radicals, they play an important role in preventing cell damage (14). A study showed that in mice fed with 10 mg/kg lead acetate, the expression of *Sod* in the olfactory bulb was significantly increased (15). On the contrary, another study observed a significant decrease in the expression of the *Sod* gene in the liver of pigs (16). In our study, the expression of all 3 *Sod*, *Gpx*, and *Cat* genes significantly decreased in exposure to Pb, which could be related to the administered dose of Pb, the animal model used, or the specific tissue examined.

In a study on *zebrafish* exposed to Pb, it was found that Pb-induced ROS stress triggered *Nrf2*, *Nqo1*, *Ho1*; downregulation of *Keap1* and altered mRNA expression of *Gpx1*, *Cu/Zn-Sod*, *Mn-Sod*, *Cyp1a*, *Ucp2* indicating involvement of Nrf2-Keap1-ARE signaling in cell defense. Nrf2-keap1 is a sensitive biomarker of Pb-induced ROS stress (17). In a study on Japanese quail (*Coturnix japonica*), cerebellar oxidative stress caused by exposure to Pb, with increased reactive oxygen radicals and malondialdehyde and Cat, Gpx, glutathione, and Sod was shown. Also, RNA-Seq analysis showed that molecular signaling pathways, especially the Nrf2/Keap1 pathway, were disrupted in the cerebellum affected by Pb (18). Heavy metals can induce ROS production in several organs. Nrf2 signaling plays a key role in maintaining antioxidant balance and can play 2 roles depending on different biological conditions. On the one hand, Nrf2 acts as an important defense mechanism against metal-induced toxicity. On the other hand, under conditions of long-term exposure and continuous activation, it may act as a catalyst in the carcinogenic process caused by metals (19). Therefore, a comprehensive knowledge of Keap1 reactivity and an understanding of how Nrf2 activity is regulated will help in the effective design of Nrf2-based targeted therapies in heavy metal toxicity.

As mentioned above, the Pb-induced toxicity mechanism is not completely known. The initial targets for the toxicity of Pb are the heme synthesis enzymes and thiol-containing enzymes (Gpx, Sod, Cat, etc.). It is supposed that Pb^2+^ could directly interfere with protein functions by binding thiol groups of cysteines (20). Our bioinformatics outcomes revealed that Res could interact with Gpx, Cat, and Sod through protein cavities near the cysteine groups. This may confirm the toxicity role of Pb against studied enzymes.

A study found that the induction of oxidative stress by the neurotoxin 1-methyl-4-phenyl-1.2.3.6-tetrahydropyridine in *Drosophila* causes H_2_O_2_ accumulation. However, the simultaneous use of neurotoxin 1-methyl-4-phenyl-1.2.3.6-tetrahydropyridine and Res significantly reduced H_2_O_2_. This also shows that Res has antioxidant properties (21). In another study, C57BL/6J mice were used to evaluate the antioxidant effect of Res. In this study, oxidative stress was induced by ethanol, and the enzymatic analysis revealed that Res increased Sod activity in HepG2 cells but had no effect on Gpx and Cat activity (22). Res significantly improves the activity of some antioxidant enzymes and damage caused by oxidative stress. However, in our study, Res also increased the expression of antioxidant genes under oxidative stress. The evaluation of molecular processes in vivo and in vitro conditions is very complicated, time and cost consuming. Our previous studies showed that bioinformatics can be a suitable tool to evaluate these processes (23). Our bioinformatics study showed that Res has binding sites on the promoter of the studied enzymes and may increase their expression in this way.

The antioxidant capacity of Res is related to the removal of ROS and its regulatory potential in the antioxidant defense of cells. However, antioxidant activities of Res are not always consistent. The effects of Res can vary depending on factors such as dose and microenvironment, and it can act as either a pro-oxidant or an antioxidant. Some studies show that Res has concentration-dependent biphasic effects. It acts as a pro-oxidant at high doses and an antioxidant at low doses both in vitro and in vivo (24). Pro-oxidant effects of Res may be followed by phosphor-PKB/Akt downregulation, cellular damage, and apoptosis (25). As an antioxidant, Res protects cells from oxidative damage by removing reactive oxygen, nitrogen species, and organic radicals. It also increases the expression of antioxidant enzymes such as Cat, Gpx, and Sod and regulates glutathione levels. These effects are carried out by regulating signaling pathways such as sirtuin1, Nrf2, and NF- κ B (26). However, we did not assess the levels of reactive oxygen and nitrogen species, which could be a limitation of our study.

Our study was conducted in 3 doses of 20, 40, and 80 mg/kg of Res. The analysis of enzyme activity and gene expression determined that the 40 mg/kg dose, and occasionally the 80 mg/kg dose, is more optimal. Various studies reported the effect of different doses of Res against heavy metal toxicity. For example, one study found that a dose of 10 mg/kg Res may protect the kidneys against oxidative damage caused by CdCl2 (27). Also, in another study, findings suggest that Res at the same dose may act as an ameliorative agent against cadmium exposure only in testicular tissue (28). In another study, a concentration of 20 mg/kg Res was used and the results showed that Res can improve the homeostatic imbalance caused by diazinon. Also, Res had a protective effect against diazinon-induced cardiotoxicity, renal, and hepatotoxicity in rats (29). Another study found that the administration of Res at a dose of 100 mg/kg can increase the liver's resistance against hepatotoxicity caused by HgCl_2_ by increasing the antioxidant efficiency (30). As can be seen, a wide range of Res has been introduced as an effective concentration in different studies. Of course, these doses can depend on the type and dose of toxic reagents, heavy metals, exposed tissue, different studied antioxidant parameters, and some other factors.

## 5. Conclusion

Res can modulate the effects of gene expression changes and antioxidant enzyme activity caused by Pb exposure in the ovaries of rats. These effects of Res are dose dependent, with the optimal dose often achieved at 40 mg/kg. However, further studies are needed to obtain more precise results.

##  Data Availability

Data supporting the findings of this study are available upon reasonable request from the corresponding author.

##  Author Contributions

H. Najafzadehvarzi and M. Karimian designed the study and conducted the research. M. Karimian, M. Ghadiri, and SM. Poormoosavi monitored, evaluated, and analyzed the results of the study. Further, M. Karimian, M. Ghadiri, SM. Poormoosavi, and H. Najafzadehvarzi reviewed the article. All authors approved the final manuscript and take responsibility for the integrity of the data.

##  Acknowledgments

This work was supported by Ayatollah Amoli Branch, Islamic Azad University, Amol, Iran. We did not use artificial intelligence in our manuscript.

##  Conflict of Interest 

The authors declare that there is no conflict of interest.
